# Research Progress on Plant RING-Finger Proteins

**DOI:** 10.3390/genes10120973

**Published:** 2019-11-26

**Authors:** Jinhao Sun, Yuhe Sun, Rana Imtiaz Ahmed, Angyan Ren, Minmin Xie

**Affiliations:** 1 Key Laboratory for Tobacco Gene Resources, Tobacco Research Institute, Chinese Academy of Agricultural Sciences, Qingdao 266101, China; 18253805901@163.com (J.S.); sunyuhe@caas.cn (Y.S.); imtiazheaven@yahoo.com (R.I.A.); ray0918@163.com (A.R.); 2 Graduate School of Chinese Academy of Agricultural Science, Beijing 100081, China; 3 Regional Agriculture Research Institute, Bahawalpur, Punjab 63100, Pakistan

**Keywords:** RING-finger proteins, adversity stress, plant development

## Abstract

E3 ubiquitin ligases are the most expanded components of the ubiquitin proteasome system (UPS). They mediate the recognition of substrates and later transfer the ubiquitin (Ub) of the system. Really Interesting New Gene (RING) finger proteins characterized by the RING domain, which contains 40–60 residues, are thought to be E3 ubiquitin ligase. RING-finger proteins play significant roles in plant growth, stress resistance, and signal transduction. In this study, we mainly describe the structural characteristics, classifications, and subcellular localizations of RING-finger proteins, as well the physiological processes of RING-finger proteins in plant growth and development. We also summarize the functions of plant RING-finger proteins in plant stress resistance. Finally, further research on plant RING-finger proteins is suggested, thereby establishing a strong foundation for the future study of plant RING-finger proteins.

## 1. Introduction

**Figure 1:**
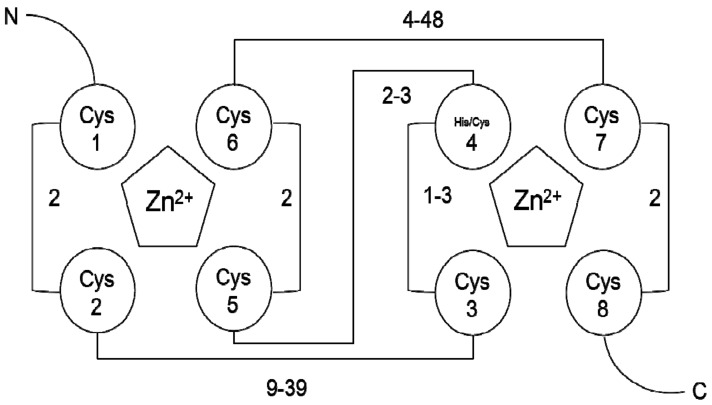
The cross structure between Really Interesting New Gene (RING)-finger protein sequences [9]. The circle represents the cysteine (Cys) and histidine (His) residue; the pentagonal form represents the bound Zn^2+^; the connecting line represents the minimum and maximum range of the number of linked amino acids; N represents the N-terminus, C represents the C-terminus, Cys1 represents ML, and Cys1 and Cys2 together with Cys5 and Cys6 bind the first Zn^2+^, whereas Cys3 and Cys4 together with Cys7 and Cys8 bind the second Zn^2+^.

Proteins diversify their functions needed for different modifications. Ubiquitin (Ub) is a protein consisting of 76 amino acids that is known as a post-translational protein modifier in all eukaryotes that affect the fate of the protein. Ub targets cellular proteins via three distinct enzymes: the Ub-activating enzyme E1, the Ub-conjugating enzyme, enzyme E2, and Ub-ligase enzyme E3 in UPS (the ubiquitin proteasome system) [1]. Ub-dependent targets are degraded specifically by the 26S proteasome based on the E3 presence, which specifically recognizes their substrates and catalyzes the isopeptide bond between substrates with Ub. Really Interesting New Gene (RING)-finger proteins, as a large family of E3 types, exist widely in eukaryotes. Previous studies have shown that RING-finger proteins are widely involved in the regulation of various physiological and biochemical processes, including plant growth and development, stress resistance, and hormone signaling responses [2,3,4,5,6]. However, compared with known DNA-binding zinc finger domains, the RING-finger domain acts as a protein–protein interaction domain [2,7] and is necessary to catalyze the E3 ligase activity of RING-finger proteins [8]. RING-finger proteins contain a conserved cysteine-rich finger domain (RING-finger Domain) consisting of 40–60 residues arranged as Cys-X_2_-Cys-X_(9-39)_-Cys-X_(1-3)_-His-X_(2-3)_-Cys/His-X_2_-Cys-X_(4-48)_-Cys-X_2_-Cys (Figure 1) [9]. It forms eight spatially conserved Cys and His residues as metal ligands (ML) to chelate two zinc atoms and define a cross-brace secondary structure that serves as a platform for binding E2s. X represents any amino acid, but the choice of these amino acids is preferred, which determines the structural and functional diversity of the family members [10]. However, in all of these loop variants, the substituted amino acids can participate in the Zn^2+^ connection, so the global three-dimensional structure of the domain is conserved [11]. These characteristics facilitate the classification of RING-finger proteins based on their domain architectures [8,9].

**Figure 2:**
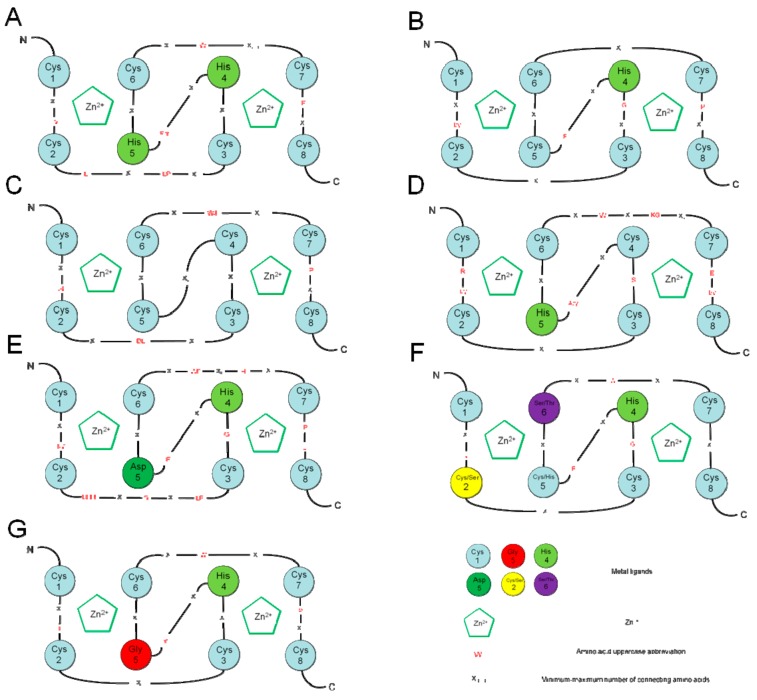
The structure diagram of different RING-finger domains. (**A**) RING-H2; (**B**), RING-HC; (**C**) RING-C2; (**D**), RING-v; (**E**) RING-D; (**F**) RING-S/T; (**G**) RING-G; N represents the N-terminus, C represents the C-terminus; 1–8 represent the ML, the blue circle represents the Cys amino acid residue, the dark green circle represents the Asp amino acid residue, the red circle represents the Gly amino acid residue, the light green circle represents the His amino acid residue, the purple circle represents the Ser/Thr amino acid residue, and and the yellow circle represents the Cys/Ser. The shape pentagons represent the Zn+; the red letter represents the conserved amino acid residue, and X stands for any amino acid.

With more extensive research, many RING-finger proteins have been identified in plants. There are 477 RING domains detected from 469 predicted proteins in the whole *Arabidopsis thaliana* proteome. These members are mainly divided into seven subtypes according to structural differences: RING-H2(241), RING-HC(186), RING-v(25), RING-C2(10), RING-D(10), RING-S/T(4), and RING-G(1). RNIG-H2 and RING-HC are two canonical subclasses of RING-finger domains, accounting for 50% and 39% of RING-finger domains, respectively [9]. However, the latest study suggests that there are 508 RING domains predicted in *Arabidopsis thaliana* due to the improved annotation of the *A. thaliana* genome. These are also divided into seven subtypes: RING-H2(258), RING-HC(191), RING-v(26), RING-C2(16), RING-D(7), RING- S/T(3), and RING-G(1) [12]. According to the type of the fifth conserved ML, the ML containing histidine is called RING-H2, and the one containing cysteine is called RING-HC. Other RING-finger types differ mainly in the spacing between the ML or the position of one or more metal ligands (Figure 2). The majority of these RING-finger proteins have been proven to possess E3 activity by ubiquitination essays *in vitro*, even the RING-finger proteins with substituted zinc binding acid residues or with slightly altered spacing [9,13,14]. A total of 425 RING-finger proteins were identified in whole rice (*Oryza sativa*) proteome and are divided into four types: RNIG-H2 (281), RING-HC (119), RING-v (23), and RING-C2 (2) [15]. Eighty percent of rice RING-finger proteins have been shown to possess E3 ligase activity [16,17]. At the same time, 731 RING-finger domains in 715 predicted proteins were divided into eight types: RING-H2 (371), RING-HCa (215), RING-HCb (47),RING-v (44), RING-C2 (38), RING-D (10), RING-S/T (5), and RING-G (1) [18]. A total of 756 RING domains in 734 predicted proteins were identified in the whole *Brassica oleracea* proteome. These domains can be further classified into eight RING types: RING-H2 (355), RING-HCa (215), RING-HCa (47),RING-v (49), RING-C2 (86), RING-D (11), RING-S/T (4), and RING-G (1) [19]. Moreover, 688 RING domains were identified from 663 predicted proteins in the whole apple (*Malus domestica*) proteome which were further divided into NINE RING types: RING-H2 (367), RING-HC (208), RING-v (35), RING-S/T (11),RING-C2(10), RING-D (1), RING-G (2), RING-mHC (44), and RING-mH2 (10) [20]. A total of 474 RING domains were identified from 469 potential proteins encoded in the *Solanum lycopersicum* genome, which are further divided into 7 RING types: RING-H2 (248), RING-HCa (142), RING-HCb (21), RING-v (40), RING-C2 (20), RING-S/T (2), and RING-G (1) [21]. In *Ostreococcus tauri*, only 65 RING domains were identified from 65 predicted proteins and further divided into 8 RING types: RING-H2 (25), RING-HC (28), RING-v (7), RING-C2 (1), C3HCHC2 (1),C2HC5 (1), C3GC3S (1), and C2SHC4 (1) [22].

## 2. RING-Finger Protein Subcellular Localization

Most of the plant RING-finger proteins are found in the nucleus, cytoplasm, and cell membrane. For example, *Arabidopsis* RFI2 is located in the nucleus [23], and rice OsCOIN is located in the nucleus and cytoplasm [24]. Meanwhile, maize ZmRFP1 is located on the cell membrane [25] (Table 1). There are also a few proteins located in the endoplasmic reticulum or other parts of the cell. RmaIH1 is located in the endoplasmic reticulum [26], and OsHCI1 is mainly distributed in the vicinity of the cytoskeleton in rice [27] (Table 1). According to recent research, the localization of RING-finger proteins is related to their function to a great extent. RING-finger proteins located in the nucleus are mainly involved in the degradation of transcription factors or other nuclear expression proteins [28,29].

However, those located in the biofilm system mainly regulates the degradation and transportation of intracellular proteins, including signal transduction components, which may be controlled by ubiquitination [30,41]. Interestingly, some RING-finger proteins can enter the nucleus from the plasma membrane to participate in the regulation of nuclear transcription factors, such as the *Arabidopsis* RGLG2 transport from the plasma membrane to the nucleus under drought stress to participate in the degradation of ERF53 [46].

## 3. RING-Finger Protein Functions

The RING-finger domains may act as a substrate binding domain [2,7], which is essential for catalyzing the E3 ligase activity of RING-finger proteins [8]. In plants, a certain number of RING-finger proteins act as E3 ubiquitin ligase. They mainly direct target proteins or interact with other proteins to participate in the gene’s expression level to regulate its various physiological processes [37].

### 3.1. RING-Finger Proteins Are Involved in Plant Growth and Development

Currently, there are few studies on RING-finger proteins involved in plant growth and development. Mainly, these studies concentrate on the role of E3 ligase in the photoperiod, leaf, and root development (Table 2).

Photomorphogenesis is critical to plant flowering. *Arabidopsis* constitutively photomorhogenic (COP1) is a negative regulator of photomorphogenesis. It directly targets the bzip transcription factor hy 5 (HY5), a positive regulator of photomorphogenesis, for degradation via the proteasome pathway in the dark [27,38]. COP1 and its interactive partner COP1 interacting protein 8 (CIP8) both possess the RING-finger domain and activity of E3 ubiquitin ligase. CIP8 may be associated with the activation of nuclear localization signals of COP1, thereby affecting the localization of COP1 in dark conditions. Moreover, CIP8 has an ubiquitin ligase function in cooperation with an E2 enzyme, AtUBC8-CIP8. It is suggested that the AtUBC8-CIP8 module can degrade HY5 in the proteasome by direct interaction with COP1 [36].

The photoperiod phenomenon is an important factor for affecting flower formation, which is the core process of plant growth and development. Red and far-red insensitive 2 (RFI2) is a RING-finger protein that participates in the photoperiod flowering pathway. The *rfi-2* promotes the expression of *CONSTANS* (*CO*), a central activator of photoperiodic flowering, and *FLOWERING LOCUS T* (*FT*) under long-day conditions, leading to an early flower phenotype. Moreover, under red and far-red light, the phenotype exhibits hypocotyl elongation [45]. However, *Arabidopsis*, a RING-finger protein with a high expression of osmotically responsive gene 1 (HOS1) (an E3 ubiquitin ligase), under intermittent cold stress treatment, leads to the degradation of CO via the ubiquitination pathway. A decrease of the CO protein leads to a delay in flowering. It was also found that HOS1 controls the transcriptional activity of phytochrome interacting factor 4 (PIF4) to participate in phyB-mediated signal light morphogenesis [42]. Another RING-finger protein heading date associated factor 1 (OsHAF1) in rice also participates in the photoperiod response process by the ubiquitination degradation of heading date 1 (HD1) [5].

RING-finger proteins are also involved in the growth and development of roots. The RING-finger drosophila protein sina (SINAT5), histone monoubiquitination1 (HUB1) in *Arabidopsis thaliana*, *Medicago sativa* RING-H2 zinc finger protein (MsRH2-1) in *M. sativa* and *Capsicum annuum* ring zinc finger protein 1 (CaRZFP1) in pepper are involved in plant root development. Overexpression of the *SINAT5* gene in Arabidopsis showed fewer lateral roots, while the lateral roots of the *sinat5* mutant plants increased. These lateral root phenotypes correlate with SINAT5, which can degrade the NAC domain containing protein 1 (NAC1) via the ubiquitin pathway [48]. The *MsRH2-1* gene is closely related to the development of lateral roots and nodules, with the highest level of transcription in roots and nodules of *M. sativa*. The MsRH2-1 overexpression line shows inhibited development of lateral roots and suggests that MsRH2-1 may function as an E3 ligase and perform a function of the E3 ligase for substrate-specific degradation via the ubiquitin-proteasome system involved in auxin signaling [73]. Overexpression of the *CaRZFP1* gene in tobacco revealed a phenotype with a larger primary root and more lateral roots in transgenic lines. CaRZFP1 was mainly related to the up-regulation of Root-hair-specific Cell Wall Proline-rich Protein (*PRP*) root hairs and lateral roots in overexpressing lines [4]. An lhk1-interacting protein (LjCZF1) from *Lotus japonicus* is a positive regulator of symbiotic nodulation, possibly through interaction with LHK1 (Lotus Histidine Kinase 1), which is essential for nodule formation [72]. However, whether CaRZFP1 and LjCZF1 are E3 ligase still needs to be verified. A mutant of the *HUB1* gene in *Arabidopsis* exhibited slower growth of primary roots. HUB1 as a RING E3 ligase regulates the growth rate of plant roots [3].

The RING-finger protein is also involved in leaf and height development. Overexpression of the *MsRH2-1* gene in alfalfa can degenerate leaves and inhibit leaf vein formation. However, overexpression of this gene in *Arabidopsis* leads to the discovery of rosettes [73]. Overexpression of the *CaRZFP1* gene in tobacco leads to fast growth, size, an increased number of leaves, and heavier fresh vegetation [4]. When the brassinosteroid-responsive RING-H2 (*BRH1*) gene in *Arabidopsis thaliana* is overexpressed, the rosette leaves of the transgenic lines were extremely curled, suggesting that it may be involved in the Brassinosteroid (BR) signaling pathway to regulate the shape of the leaves [35]. However another RING finger protein, (the endoplasmic reticulum-associated degradation) ERAD-mediating RING finger protein (EMR), an E3 ligase, also affects the synthesis of BR signaling proteins on the endoplasmic reticulum to regulate plant height [40]. Silencing of the *Nicotiana benthamiana* zinc finger (NbZFP1) hampered fruit development. Compared to the WT (wild type), overexpression of *NbZFP1* displayed a short internode length and a sturdy stem phenotype [75].

RING-finger proteins are also involved in fruit development. Flying saucer1 (FLY1) and big brother (BB) are used as E3 ligases for floral organs of the seed pectins and control the degree of methyl esterification. However, the regulation mechanisms are not clear for these two proteins [34,41]. It is believed that with continuous research, the mechanism of the RING-finger protein involved in the growth and development process will be more clearly revealed.

### 3.2. RING-Finger Proteins Are Involved in Plant Stress Resistance

Stress is an important limiting factor affecting plant growth and crop production. Over the long process of evolution, plants have produced many different biotic/abiotic stress responses and regulation methods. In recent years, many studies have shown that RING finger proteins are involved in responding to biotic and abiotic stress in plants (Table 3).

#### 3.2.1. RING-Finger Proteins Are Involved in Plant Drought Resistance

Drought is a major abiotic stress factor affecting plant survival. It is necessary to analyze drought resistance genes and analyze their drought resistance mechanisms. Recently, it has been reported that several RING-containing proteins function as E3 ligases in response to the Abscisic Acid (ABA) dependent defense mechanism against drought stress. A study found that *XERICO* in *Arabidopsis*, and its homologous genes *ZmXerico1/2* in *Zea mays*, are overexpressed in *Arabidopsis* and improve resistance to drought. However, *XERICO* raises the biosynthesis of ABA by degrading the ASK1-interacting F-box protein (AtTLP9) in the proteasome system, while ZmXerico1/2 makes the ABA more stable via the ubiquitin of ABA 8′-hydroxylases to improve drought resistance [50,64]. In the rice *osdsg1* mutant line and Delayed Seed Germination 1 (*OsDSG1)-RNAi* plants, the expression level of ABA signaling-related genes was significantly increased and lead to greater resistance to drought than the wild-type. It was estimated that OsDSG1 degrades aba insensitive 3 (OsAIB3) via the 26S proteasome system and negatively regulates plant drought resistance by participating in the ABA-pathway [53]. The *Capsicum annuum* ADIP1 interacting ring finger protein 1 (CaAIRF1) degrades *Capsicum annuum* Type 2C Protein Phosphatase (CaADP1) through the ubiquitination pathway by changing the sensitivity to ABA to improve drought resistance [61]. The *Capsicum annuum* drought sensitive RING finger protein 1(CaDSR1) exhibited E3 ligase activity and promoted CaDILZ1 expression through the 26S proteasome pathway to alter ABA content in the modulation of drought tolerance [63]. *Capsicum annuum* ABA sensitive RING finger E3 ligase 1 (CaASRF1) positively modulates ABA signaling via modulation of CaAIBZ1’s stability to drought stress [62]. Many RING genes in different species are induced by ABA to respond to drought stress, such as *Arabidopsis* ABA-insensitive RING protein 1 (*AtAIRP1*), *AtAIRP4*, *Nicotiana tabacum RING-H2* Finger Gene 1 (*NtRHF1*), *OsBIRF1*, *TaRZF70,* and *AdZFP1* [30,31,51,68,69,76]. Furthermore, AtAIRP1 and NtRHF1 have been proven to be E3 ligases. However, OsBIRF1, TaRZF70, and AdZFP1 have not been proven to be E3 ligases. Some RING-finger protein independent ABA-pathways also respond to drought stress responses. DREB2A-interacting protein1 (DRIP1) and DRIP2, isolated from *Arabidopsis thaliana,* acts as a negative regulator in drought-responsive gene expression by targeting dehydration-responsive element binding protein 2 (ADREB2A) to facilitate 26S proteasome proteolysis [39]. NUCLEAR FACTORY A 5 (NFY5), a key drought-induced transcription factor, can be degraded by NFYA5 enhancing RING finger (NERF) in the proteasome pathway, which is important for controlling stomatal closure and drought resistance in *Arabidopsis thaliana* [28]. RING membrane-anchor 1 (Rma1) in Arabidopsis thaliana, and its homologous Rma1H1 in pepper, can mediate the ubiquitination of plasma membrane aquaporin (PIP2), which positively regulates plant drought resistance [26]. RING domain ligase 2 (RGLG2) negatively regulates drought stress response via the ubiquitin ethylene response factor 53 (AtERF53) in Arabidopsis [46]. The *Oryza sativa* RING domain-containing protein OsRDCP1, as a RING E3 ligase, may be involved in the transportation or degradation of a negative transcription factor or factors that inhibit(s) the expression of water stress-induced genes [56]. The *Oryza sativa* drought-induced SINA protein OsDIS1, via the 26S proteasome-dependent pathway, degrades *Oryza sativa* NIMA-related kinase 6 (OsNek6), plaing a negative role in drought stress tolerance. The orthologue protein in wheat (*Triticum aestivum L*.) TaDIS1 may perform a negatively function in drought stress by regulating the stress response-related genes [52,67].

#### 3.2.2. RING-Finger Proteins Are Involved in Salt and Aluminium Resistance

Some RING-finger proteins are also involved in salt stress. The salt and drought-induced ring finger 1 (SDIR1) in *Arabidopsis thaliana* and its homologous protein ZmRFP1 are found to participate in the regulation of drought’s stress response. However, SDIR1 also acts as an E3 ligase to ubiquitinate the modification of SDAIR-interacting protein 1 (SDIR1P1) to regulate the expression of transcription factor *ABA-INSENSITIVE5* (*ABI5*), a key ABA-pathway gene, thereby participating in the salt response process of *Arabidopsis thaliana* [47]. *Oryza sativa* salt, ABA, and drought stress-induced RING finger protein 1 (OsSADR1) act as E3 ligases and function negatively in drought and salt stress [58]. CaRFP1 in pepper is an E3 ligase and directly targets the basic PR-1 protein (CaBPR1) to ubiquitinate the modification involved in the signaling pathway of ABA in response to salt stress [13]. Salt tolerance RING finger 1 (STRF1) from *Arabidopsis* and *Oryza sativa* salt-induced RING finger protein 1 (OsSIRP1), as E3 ligases, participate in the response of salt stress. STRF1 mainly regulates the expression of membrane transport-related proteins. OsSIRP1 is a negative regulator of salt tolerance, and its target protein needs to be further studied [49,60]. The microtubule-associated RING finger protein 1 (OsMAR1), an E3 ligase, acts as a negative regulator for salt-stress response through the regulation of the *O. sativa* chymotrypsin protease inhibitor 2 (OsCPI2), but anther rice RING H2-type E3 ligase, OsSIRH2-14 (previously named OsRFPH2-14), plays a positive role in salinity tolerance by regulating salt-related proteins, including an HKT-type Na+ transporter (OsHKT2; 1) [55,59]. The RING-finger proteins *M. esculenta* RZF (MeRZF) and SpRing were found to respond to salt stress in cassava and wild tomato, respectively. However, the mechanism by which they play a role remains to be further studied [74,77].

Aluminum (Al) toxicity is a major limiting factor in the production of acid soil crops. In recent studies, a series of E3 ubiquitin ligases have been discovered to regulate plant Al tolerance or resistance. *Arabidopsis thaliana* Al tolerance RING finger 1 (AtATRF1), acting as an E3 ligase, mediates the aluminum tolerance of *Arabidopsis thaliana*. Studies have shown that plants overexpressing AtATRF1 enhance tolerance to Al, while Al can induce the expression of AtATRF1. AtATRF1 is located in the nucleus and may interact and ubiquitinate Ataxia telangiectasia-mutated and RAD3-like Protein (AtATR), a transcriptional regulator, which plays an important role in plant growth and development [32]. Soybean ariadne-like ubiquitin ligase protein (GmARI1) functions as an E3 ligase and might mediate soybean responses to the tolerance of Al stress through oxidative species signals, which may overlap with plant hormone signaling pathways [71].

#### 3.2.3. RING-Finger Proteins Are Involved in Temperature Stress

Low-temperature or high-temperature stress affect the normal life metabolism of plants, thereby affecting their growth and development. Recently, there have been many reports about RING-finger proteins in response to low-temperature stress. The RING-finger protein HOS1 in *Arabidopsis thaliana* acts as a negative regulator of low-temperature response gene transcription and can function as an E3 ubiquitin ligase. Under low temperature conditions, HOS1 can degrade an inducer of cbf expression 1 (ICE1) through the 26S protease pathway, while overexpression of HOS1 makes plants sensitive to low temperatures [43,78]. *Oryza sativa* cold-inducible (OsCOIN) could respond to low-temperature stress, relying on the ABA pathway [24]. Furthermore, the expression of cold stress-related genes, such as *OsLti6b* and *OsP5CS*, could be induced in the overexpression line of *OsCOIN*, making it useful for enhancing tolerance to low temperatures [24]. The expression of the *BrRZFP* gene in *Brassica rapa* can be induced by low temperatures; the ABA, drought, and salt stress resistance of tobacco plants heterologously expressing this gene are also enhanced [70]. However, OsCOIN and BrRZFP have not been shown to function as E3 ligases. There are few reports on the response of RING finger proteins, which are mainly concentrated in rice, to high temperature stress. *Oryza sativa* heat and cold induced 1(OsHCI1) and *Oryza sativa* heat-induced RING finger protein 1(OsHIRP1) both act as E3 ligases to positively regulate heat stress responses [27,29]. The rice *OsRZFP34* gene and *HEAT TOLERANCE AT SEEDLING STAGE* (*OsHTAS*) gene can participate in the ABA pathway, change the stomatal switch state in leaves, and improve high-temperature tolerance. However, only OsHTAS was proven to be an E3 ligase [54,57].

#### 3.2.4. RING-Finger Proteins Are Involved in Biotic Stress

Biotic stress is general term for various biological factors that are unfavorable to survival and development, including pests, fungi, bacteria, and viruses. Recent studies have shown that RING-finger proteins are involved in biotic stress responses in many species. The RING-finger protein MYB30-interacting E3 ligase 1 (MIEL1) in *Arabidopsis thaliana* acts as an E3 ligase ubiquitinating transcription factor Myb domain protein 30 (MYB30) and degrades transgenic MYB30, thereby reducing the expression of disease-resistant genes and reducing the plant’s immune response [44]. *Arabidopsis* toxicos en levadura 9 (ATL9) is also involved as an E3 ligase in regulating plant resistance to viable nutrient pathogens. When a pathogen infects a plant, it can induce the expression of *ATL9* and ATL9 to resist the inhibitory protein of the pathogen by ubiquitination hydrolysis, thereby promoting an immune response [33]. When the *CaRFP1* gene was overexpressed in *Arabidopsis thaliana*, the transgenic plants became more sensitive to tomato bacterial spot disease. This may be due to the fact that the protein acts as an E3 ligase, degrading the expression levels of disease-related genes such as (synthesis of pathogenesis-related) *PR-2* and *PR-5* [13]. The Erysiphe necator-induced RING finger protein 1 (EIRP) is also involved in the pathogen defense response in *Vitis pseudoreticulata* by degrading the transcription factor VpWRKY11 through the ubiquitin proteasomal system, which enhances the ability of East China Grapes to resist pathogens [65]. Another RING finger protein in *Vitis pseudoreticulata* VpRH2, as an E3 ligase, improves resistance to powdery mildew by interacting with VpGRP2A [66]. Heterologous expression of the *OsBIRF1* gene and overexpression of the *NbZFP1* gene in tobacco could enhance resistance to the tobacco mosaic virus, and these two RING-finger proteins may enhance disease resistance by regulating the expression of PR genes [75].

## 4. Conclusions

The growth and development of plants and their ability to adapt to a variety of stresses are mainly realized by changing their protein expression and metabolic pathways. It is of great significance to study the expression and function of these proteins in order to improve plant growth and tolerance to stress [79,80,81]. Protein ubiquitination is one of the most important modifications after protein translation in plants, and the ubiquitin ligase E3 determines the specific selection of substrate proteins. Our study demonstrates that the plant RING-finger family of E3 ligases is quite diverse. In addition to the previously defined types of RING-finger domains, the types we identified include modified RING-finger domains that display variation in spacing between, or have amino acids substitutions at, conserved zinc-coordinating residues. Therefore, searches for RING-finger proteins should not be limited to known types. However, a greater characterization of plant RING-finger domain types is needed to further define the requirements for functional RING-finger proteins. The presence of various types of RING-finger domains are involved in the ubiquitination pathway, and each type of RING-finger domain may correspond to multiple E2 enzymes. Further biochemical analyses utilizing different families of plant E2s to define functional E2–E3 combinations would give insight into the specific requirements for E2–E3 interactions. The number of RING-finger proteins, the different types of RING-finger domains, and the presence of a variety of protein–protein interaction domains in the RING-finger proteins suggest a role for the RING-type E3 ligase in different cellular processes via the targeted regulation of numerous substrates. At present, RING-finger protein research is mainly focused on plant growth and development, as well as related studies. These RING-fingers are mainly used as ubiquitin ligase E3 to degrade other proteins through the 26 proteasomes. Most of them are involved in the ABA pathway and participate in anti-stress, such as XERICO and CaDSR1 [50,63]. However, it remains to be determined how E3 proteins are interacted with substrate proteins, whether they recognize substrate proteins with the same characteristics, and whether E3 ubiquitin ligase can modify substrate proteins by ubiquitin or poly-ubiquitin. In addition, current research is mainly concentrated in Arabidopsis and rice, while little is known about other species, such as wheat and corn. The function of a large number of RING-finger proteins still needs to be discovered and studied in different species. With the continuous development of genome sequencing technology, more plant RING-finger proteins will be identified, which will be comprehensively studied by bioinformatics analysis, functional genomics, transcriptomics, proteomics, and metabolomics. This will greatly facilitate the study of the function and mechanism of RING-fingers and accelerate the process of genetic engineering to create excellent new germplasms.

## Figures and Tables

**Table 1:** The subcellular localizations of RING-finger proteins.

Gene Name	Plant Species	Type	Subcellular Localization	References
*AtAIRP1*	*A. thaliana*	RING-HC	Cell membrane	Ryu M.Y. et al., 2010 [30]
*AtAIRP4*	*A. thaliana*	RING-HC	Cytoplasm	Yang L. et al., 2016 [31]
*AtATRF1*	*A. thaliana*	RING-HC	Nucleus	Qin X. et al., 2017 [32]
*ATL9*	*A. thaliana*	RING-HC	Endoplasmic network	Beerocal M. et al., 2010 [33]
*BIG BROTHER*	*A. thaliana*	RING-H2		Dish S. et al., 2006 [34]
*BRH1*	*A. thaliana*	RING-H2		Wang X. et al., 2018 [35]
*CIP8*	*A. thaliana*	RING-H2		Hardtke C.S. et al., 2002 [36]
*COP1*	*A. thaliana*	RING-HC	Nucleus	Deng X.W. et al., 1991, Von Arnim A.G. et al., 1993 [37,38]
*DRIP1*	*A. thaliana*	RING-HC	Nucleus	Qin F. et al., 2008 [39]
*DRIP2*	*A. thaliana*	RING-HC	Nucleus	Qin F. et al., 2008 [39]
*EMR*	*A. thaliana*	RING-HC	Cytoplasm	Park J.H. et al., 2018 [40]
*FLY1*	*A. thaliana*	RING-H2	Golgi apparatus	Voiniciuc C. et al., 2013 [41]
*HOS1*	*A. thaliana*	RING-C2	Nucleus	Lee H. et al.,2001, Dong C.H. et al., 2006, Kima J.H. et al., 2017 [42,43]
*HUB1*	*A. thaliana*	RING-HC		Fleury D. et al., 2007 [3]
*MIEL1*	*A. thaliana*	RING-H2	Nucleus	Marino D. et al., 2013 [44]
*NERF*	*A. thaliana*	RING-HC	Nucleus	Gao W. et al., 2015 [28]
*RFI2*	*A. thaliana*	RING-H2	Nucleus	Chen M.J. et al., 2006 [45]
*RGLG2*	*A. thaliana*	RING-HC	Cell membrane	Cheng M.C. et al., 2012 [46]
*Rma1*	*A. thaliana*	RING-HC	Endoplasmic network	Lee H.K. et al., 2009 [26]
*SDIR1*	*A. thaliana*	RING-H2	Cell membrane	Zhang Y.Y. et al., 2007 [47]
*SINAT5*	*A. thaliana*	RING-HC	Nucleus	Xie Q. et al., 2002 [48]
*STRF1*	*A. thaliana*	RING-H2	Cytoplasm and cell membrane	Tian M.M. et al., 2015 [49]
*XERICO*	*A. thaliana*	RING-H2		Ko J.H. et al., 2006 [50]
*OsBIRF1*	*O. sativa*	RING-H2		Liu H.Z. et al., 2008 [51]
*OsCOIN*	*O. sativa*	RING-C2	Nuclear and cytoplasm	Liu K.M. et al., 2007 [24]
*OsDIS1*	*O. sativa*	RING-H2	Nucleus	Ning Y. et al., 2011 [52]
*OsDSG1*	*O. sativa*	RING-H2		Park G.G. et al., 2010 [53]
*OsHAF1*	*O. sativa*	RING-HC		Yang Y. et al., 2015 [5]
*OsHCI1*	*O. sativa*	RING-HC	Golgi apparatus	Lim S.D. et al., 2013 [27]
*OsHIRP1*	*O. sativa*	RING-HC	Nucleus	Kim J.H. et al., 2019 [29]
*OsHTAS*	*O. sativa*	RING-H2	Nuclear and cytoplasm	Liu J.P. et al., 2016 [54]
*OsMAR1*	*O. sativa*	RING-H2	Related to the microtubules	Park Y.C. et al., 2018 [55]
*OsRDCP1*	*O. sativa*	RING-HC	Cell membrane	Bae H. et al., 2011 [56]
*OsRZFP34*	*O. sativa*	RING-HC		Hus K.H. et al., 2014 [57]
*OsSADR1*	*O. sativa*	RING-H2	Nucleus	Hwang S.G. et al., 2018 [58]
*OsSIRH2-14*	*O. sativa*	RING-H2	Cell membrane, cytoplasm, Golgi	Park Y.C. et al., 2019 [59]
*OsSIRP1*	*O. sativa*	RING-HC	Cytoplasm	Hwang S.G. et al., 2016 [60]
*CaAIRF1*	*C. annuum L.*	RING-HC	Nucleus	Lim C.W. et al., 2017 [61]
*CaASRF1*	*C. annuum L.*	RING-H2	Nuclear and cytoplasm	Joo H. et al., 2019 [62]
*CaDSR1*	*C. annuum L.*	RING-H2	Nuclear and cytoplasm	Lim C.W. et al., 2018 [63]
*CaRFP1*	*C. annuum L.*	RING-HC		Hong J.K. et al., 2007 [13]
*CaRZFP1*	*C. annuum L.*	RING-HC		Zeba N. et al., 2009 [4]
*RmaIH1*	*C. annuum L.*	RING-HC	Endoplasmic network	Lee H.K. et al., 2009 [26]
*ZmRFP1*	*Z. mays L.*	RING-H2	Cell membrane	Xia Z.L. et al., 2012 [25]
*ZmXerico1*	*Z. mays L.*	RING-H2	Cytoplasm	Brugière N. et al., 2017 [64]
*ZmXerico2*	*Z. mays L.*	RING-H2	Cytoplasm	Brugière N. et al., 2017 [64]
*EIRP1*	*V. pseudoreticulata*	RING-HC	Nucleus	Yu Y. et al., 2013 [65]
*VpRH2*	*V. pseudoreticulata*	RING-H2	Cytoplasm and cell membrane	Wan G.L. et al., 2017 [66]
*TaDIS1*	*T. aestivuml*	RING-HC		Liu Y. et al., 2018 [67]
*TaRZF70*	*T. aestivuml*	RING-H2		Kam J. et al., 2007 [68]
*AdZFP1*	*A. dracunculus L.*	RING-HC		YanG.X. et al., 2008 [69]
*BrRZFP1*	*B. rapa*	RING-HC	Cytoplasm and cell membrane	Jun Y.J. et al., 2013 [70]
*GmARI1*	*G. max*	*RING-HC*	Nucleus	Zhang X.L. et al., 2014 [71]
*LjCZF1*	*L. japonicus*	*RING-HC*	Cytoplasm and cell membrane	Cai K. et al., 2018 [72]
*MsRH2-1*	*M. sativa*	*RING-H2*		Karlowski W.M. et al., 2003 [73]
*MeRZF*	*M. esculenta*	*RING-H2*	Cell membrane	Reis S.P.D. et al., 2012 [74]
*NbZFP1*	*N. benthamiana*	*RING-HC*	Chloroplast	Wu W.X. et al., 2014 [75]
*NtRHF1*	*N. tabacum*	*RING-H2*		Xia Z.L. et al., 2012 [76]
*SpRing*	*S. lycopersicum*	*RING-H2*	Endoplasmic network	Qi S.L. et al., 2016 [77]

**Table 2:** RING-finger proteins involved in plant growth and development.

Gene Name	AGI Loci	Protein	Function	References
*BIG BROTHER*	AT3g63530	E3 ligase	regulator of *Arabidopsis* floral organ size	Dish S. et al., 2006 [34]
*BRH1*	AT3g61460	E3 ligase	alters rosette leaf shape	Wang X. et al., 2018 [35]
*CIP8*	AT5g64920	E3 ligase	regulator of photomorphogenesis	Hardtke C.S. et al., 2002 [36]
*COP1*	AT2g32950	E3 ligase	regulator of photomorphogenesis	Von Arnim A.G. et al., 1993 [38]
*CaRZFP1*			regulator of root development	Zeba N. et al., 2009 [13]
*EMR*	AT4g26400	E3 ligase	involved in the degradation of ER-associated protein	Park J.H. et al., 2018 [40]
*FLY1*	AT4g28370	E3 ligase	regulates the degree of pectin methylesterification in seed mucilage	Voiniciuc C. et al., 2013 [41]
*HOS1*	AT2g39810	E3 ligase	regulator of photomorphogenesis and flowering time	Lee H. et al. 2001, Kima J. Het al., 2017 [42,43]
*HUB1*	AT2g44950	E3 ligase	regulator of root development	Fleury D. et al., 2007 [3]
*LjCZF1*		E3 ligase	a positive regulator of symbiotic nodulation	Cai K. et al., 2018 [72]
*MsRH2-1*			regulator of root and nodule development	Karlowski W.M. et al., 2003 [73]
*NbZFP1*			regulator of fruit development, plant height, and leaf spacing	Wu W.X. et al., 2014 [75]
*OsHAF1*		E3 ligase	regulator of photomorphogenesis	Park G.G. et al., 2010 [5]
*RFI2*	AT2g47700	E3 ligase	regulator of flowering time	Chen M.J. et al., 2006 [45]
*SINAT5*	AF480944	E3 ligase	regulator of lateral root development	Xie Q. et al., 2002 [48]

**Table 3:** RING-finger proteins involved in plant stress resistance.

Gene Name	AGI Loci	Protein	Function	References
*AdZFP1*			regulator of plant tolerance to drought stress	Yang X. et al., 2008 [69]
*AtAIRP1*	AT4G23450	E3 ligase	regulator of plant tolerance to drought stress	Ryu M.Y. et al., 2010 [30]
*AtAIRP4*	AT5G58787	E3 ligase	regulator of plant tolerance to drought stress	Yang L. et al., 2016 [31]
*AtATRF1*		E3 ligase	regulator of plant tolerance to drought stress	Qin X. et al., 2017 [32]
*ATL9*	AT2g35000	E3 ligase	regulator of plant resistance to viable nutrient pathogens	Beerocaccobo M. et al., 2010 [33]
*BrRZFP1*			regulator of plant tolerance to drought and cold stress	Jung Y.J. et al., 2013 [70]
*CaAIRF1*		E3 ligase	regulator of plant tolerance to drought stress	Lim C.W. et al., 2017 [61]
*CaASRF1*		E3 ligase	regulator of plant tolerance to drought stress	Joo H. et al., 2019 [62]
*CaDSR1*		E3 ligase	regulator of plant tolerance to drought stress	Lim C.W. et al., 2018 [63]
*CaRFP1*		E3 ligase	regulator of plant tolerance to salt stress	Hong J.K. et al., 2007 [13]
*DRIP1*		E3 ligase	regulator of plant tolerance to drought stress	Qin F. et al., 2008 [39]
*DRIP2*		E3 ligase	regulator of plant tolerance to drought stress	Qin F. et al., 2008 [39]
*EIRP1*		E3 ligase	involved in pathogen defense	Yu Y. et al., 2013 [65]
*GmARI1*		E3 ligase	regulator of plant tolerance to Aluminium stress	Zhang X.L. et al., 2014 [73]
*HOS1*	AT2G39810	E3 ligase	regulator of plant tolerance to cold stress	Dong C.H. et al., 2006 [78]
*MeRZF*			regulator of plant tolerance to salt stress	Reis S.P.D. et al., 2012 [74]
*MIEL1*	AT5G18650	E3 ligase	regulator of plant tolerance to biotic stress	Marino D. et al., 2013 [44]
*NbZFP1*			regulator of plant tolerance to tobacco mosaic virus	Wu W.X. et al., 2014 [75]
*NERF*		E3 ligase	regulator of plant tolerance to drought stress	Gao W. et al., 2015 [28]
*NtRHF1*		E3 ligase	regulator of plant tolerance to drought stress	Xia Z.L. et al., 2012 [76]
*OsBIRF1*			regulator of plant tolerance to drought stress	Liu H.Z. et al., 2008 [51]
*OsCOIN*			regulator of plant tolerance to cold stress	Liu K.M. et al., 2007 [24]
*OsDSG1*		E3 ligase	regulator of plant tolerance to drought stress	Liu K.M. et al., 2007 [53]
*OsHCI1*		E3 ligase	regulator of plant tolerance to high temperature stress	Lim S.D. et al., 2013 [27]
*OsHIRP1*		E3 ligase	regulator of plant tolerance to high temperature stress	Kim J.H. et al., 2019 [29]
*OsHTAS*		E3 ligase	regulator of plant tolerance to high temperature stress	Liu J.P. et al., 2016 [54]
*OsMAR1*		E3 ligase	regulator of plant tolerance to salt stress	Park Y.C. et al., 2018 [55]
*OsRDCP1*		E3 ligase	regulator of plant tolerance to drought stress	Bae H. et al., 2011 [56]
*OsRZFP34*			regulator of plant tolerance to high temperature stress	Hus K.H. et al., 2014 [57]
*OsSADR1*		E3 ligase	regulator of plant tolerance to drought stress	Hwang S.G. et al., 2018 [58]
*OsSIRH2-14*		E3 ligase	regulator of plant tolerance to salt stress	Park Y.C. et al., 2019 [59]
*OsSIRP1*		E3 ligase	regulator of plant tolerance to salt stress	Hwang S.G. et al., 2016 [60]
*RGLG2*	AT5G14420	E3 ligase	regulator of plant tolerance to drought stress	Cheng M.C. et al., 2012 [46]
*Rma1*	AT4G03510	E3 ligase	regulator of plant tolerance to drought stress	Lee H.K. et al., 2009 [26]
*RmaIH1*		E3 ligase	regulator of plant tolerance to drought stress	Lee H.K. et al., 2009 [26]
*SDIR1*	AT3G55530	E3 ligase	regulator of plant tolerance to salt stress	Lee H.K. et al., 2009 [47]
*SpRing*			regulator of plant tolerance to salt stress	Qi S.L. et al., 2016 [77]
*STRF1*		E3 ligase	regulator of plant tolerance to salt stress	Tian M.M. et al., 2015 [49]
*TaDIS1*			regulator of plant tolerance to drought stress	Liu Y. et al., 2018 [67]
*TaRZF70*			regulator of plant tolerance to drought stress	Kam J. et al., 2007 [68]
*VpRH2*	KU296022		improves resistance to powdery mildew fungus	Wang L. et al., 2017 [66]
*XERICO*	AT2G04240	E3 ligase	regulator of plant tolerance to drought stress	Ko J.H. et al., 2006 [50]
*ZmRFP1*		E3 ligase	regulator of plant tolerance to drought stress	Xia Z.L. et al., 2012 [25]
*ZmXerico1*		E3 ligase	regulator of plant tolerance to drought stress	Brugière N. et al., 2017 [64]
*ZmXerico2*		E3 ligase	regulator of plant tolerance to drought stress	Brugière N. et al., 2017 [64]
